# Leucine-Rich Repeat Kinase 2 (LRRK2) phosphorylates p53 and induces p21^WAF1/CIP1^ expression

**DOI:** 10.1186/s13041-015-0145-7

**Published:** 2015-09-18

**Authors:** Dong Hwan Ho, Hyejung Kim, Jisun Kim, Hyuna Sim, Hyunjun Ahn, Janghwan Kim, Hyemyung Seo, Kwang Chul Chung, Bum-Joon Park, Ilhong Son, Wongi Seol

**Affiliations:** InAm Neuroscience Research Center, Sanbon Medical Center, College of Medicine, Wonkwang University, 321 Sanbon-ro, Gunposhi Gyeonggido, Republic of Korea; Department of Molecular and Life Sciences, Hanyang University, Ansanshi, Gyeonggido, Republic of Korea; Stem Cell Research Center, Korea Research Institute of Bioscience and Biotechnology (KRIBB), 125 Gwahak-ro, Yuseong-gu, Daejeon Republic of Korea; Korea University of Science & Technology (UST), 113 Gwahak-ro, Yuseong-gu, Daejeon Republic of Korea; Department of Systems Biology, College of Life Science and Biotechnology, Yonsei University, Seodaemun-gu, Seoul Republic of Korea; Department of Molecular Biology, College of Natural Science, Pusan National University, Pusan, Republic of Korea; Department of Neurology, Sanbon Medical Center, College of Medicine, Wonkwang University, 321 Sanbon-ro, Gunposhi, Gyeonggido Republic of Korea

## Abstract

**Background:**

Leucine-rich repeat kinase 2 (LRRK2) is a gene in which a mutation causes Parkinson’s disease (PD), and p53 is a prototype tumor suppressor. In addition, activation of p53 in patient with PD has been reported by several studies. Because phosphorylation of p53 is critical for regulating its activity and LRRK2 is a kinase, we tested whether p53 is phosphorylated by LRRK2.

**Results:**

LRRK2 phosphorylates threonine (Thr) at TXR sites in an *in vitro* kinase assay, and the T304 and T377 were identified as putative phosphorylated residues. An increase of phospho-Thr in the p53 TXR motif was confirmed in the cells overexpressing G2019S, and human induced pluripotent stem (iPS) cells of a G2019S carrier. Interactions between LRRK2 and p53 were confirmed by co-immunoprecipitation of lysates of differentiated SH-SY5Y cells. LRRK2 mediated p53 phosphorylation translocalizes p53 predominantly to nucleus and increases p21^WAF1/CIP1^ expression in SH-SY5Y cells based on reverse transcription-polymerase chain reaction and Western blot assay results. The luciferase assay using the p21^WAF1/CIP1^ promoter-reporter also confirmed that LRRK2 kinase activity increases p21 expression. Exogenous expression of G2019S and the phosphomimetic p53 T304/377D mutants increased expression of p21^WAF1/CIP1^ and cleaved PARP, and cytotoxicity in the same cells. We also observed increase of p21 expression in rat primary neuron cells after transient expression of p53 T304/377D mutants and the mid-brain lysates of the G2019S transgenic mice.

**Conclusion:**

p53 is a LRRK2 kinase substrate. Phosphorylation of p53 by LRRK2 induces p21^WAF1/CIP1^ expression and apoptosis in differentiated SH-SY5Y cells and rat primary neurons.

**Electronic supplementary material:**

The online version of this article (doi:10.1186/s13041-015-0145-7) contains supplementary material, which is available to authorized users.

## Background

Leucine-rich repeat kinase 2 (*LRRK2*) is one of the Parkinson’s disease (PD)-causative genes along with *SNCA* (α-synuclein), *PARK2* (Parkin), *DJ*-*1* and *PINK1* [1, 2]. LRRK2 contains both kinase and GTPase domains [3–5] whose activities are critical for signal transduction. Moreover, the G2019S pathogenic mutation [6] increases kinase activity [7, 8] and affects various pathogenic phenotypes such as increased neuronal cytotoxicity and protein aggregation [7, 9, 10], decreased neurite length [11, 12] and changes in the autophagy rate [12, 13]. In addition, pharmacological inhibition of kinase activity rescues pathogenic phenotypes, such as neurocytotoxicity and defective neurite outgrowth [14]. Because of the relevance of LRRK2 kinase activity to PD pathogenesis, LRRK2 has emerged as a therapeutic target of PD, and the effort to identify LRRK2 kinase substrates and chemical inhibitors has intensified [15–21]. To date, several diverse proteins have been reported as potential LRRK2 kinase substrates. Some examples are β-tubulin [22], ArfGAP1 [23, 24], tau [25], members of the mitogen-activated protein kinase kinase family [26], eukaryotic initiation factor 4E-binding protein (4E-BP; [27]), Akt1 [28], ribosomal protein s15 [19], endophilin A [18], Rab5 [21], Bcl-2 [29] and Snapin [20]. It is unclear whether these proteins are actual physiological substrates. Some studies suggest that LRRK2 plays roles in vesicle trafficking, autophagy, mitochondrial dysfunction, and inflammation [13, 18, 30–32]. In addition, one study identified several ribosomal proteins as LRRK2 kinase substrates using an unbiased proteomics suggesting that LRRK2 is a translational regulator [19] along with a previous 4E-BP study [27].

p53 is encoded by *TP53*, and is a well-known tumor suppressor that is mutated in numerous types of tumor cells [33]. p53 is a transcription factor that is degraded through interacting with MDM2 under normal condition, but p53 is phosphorylated at specific residues, become stabilized and resistant to its own degradation during exposure to genomic or other stressors [34, 35]. Phosphorylated p53 enhances its nuclear retention, resulting in increased p53 binding to its target sequences and activation of target genes, most of which function in apoptosis or cell growth arrest [36].

p53 has been postulated to contribute to PD pathogenesis. For example, PD toxins, such as MPTP, rotenone, and 6-hydroxydopamine activate p53 [37]. In addition, PD causative genes, such as Parkin, DJ-1, and α-synuclein affect p53 expression or its activity [37–39].

Here, we report that LRRK2 phosphorylates p53, which could be another clue to the relationship between p53 and PD pathogenesis.

## Results

### LRRK2 phosphorylates p53 *in vitro*

**Fig. 1:**
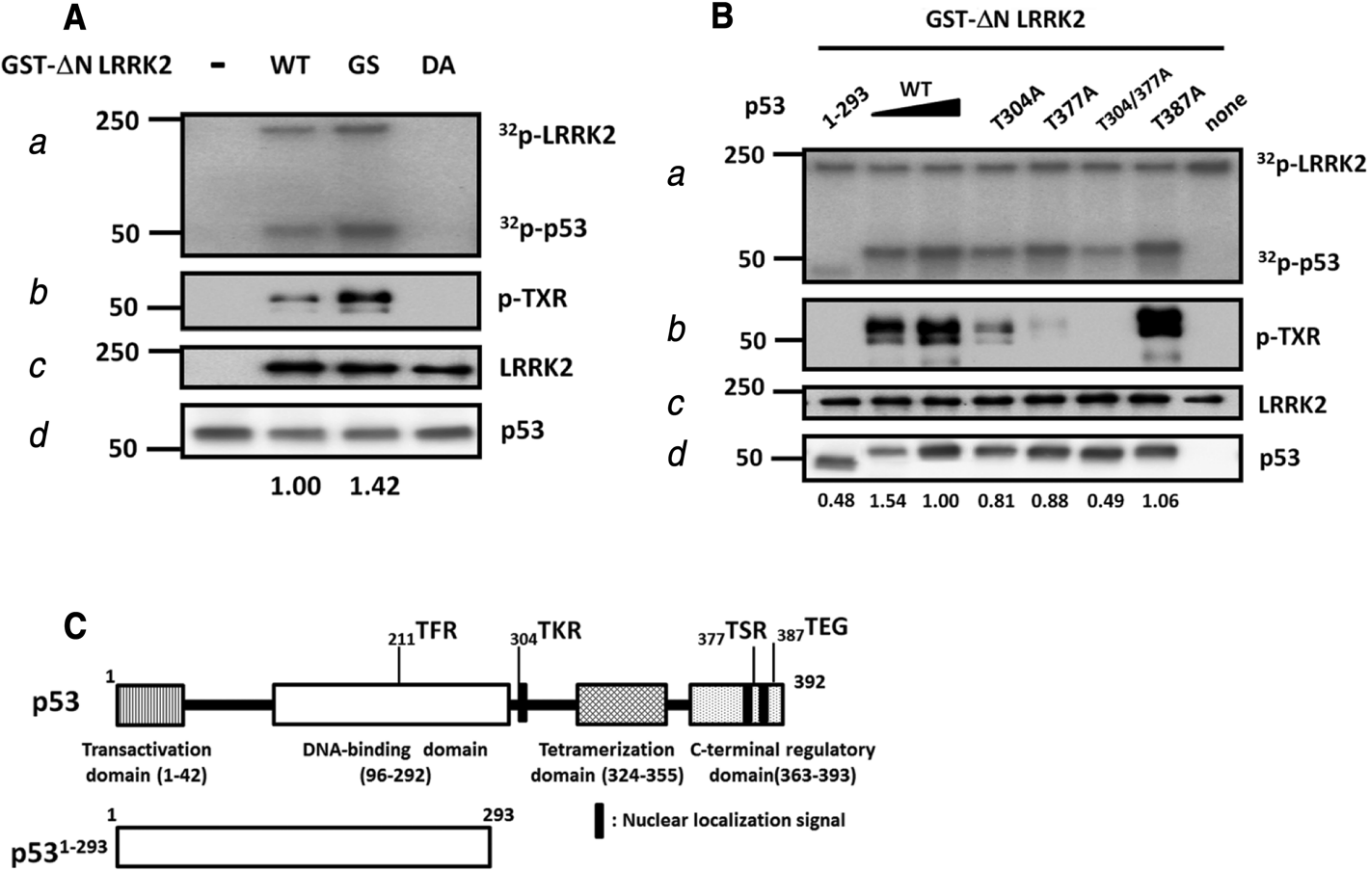
LRRK2 phosphorylates p53 at T304 and T377 in an *in vitro* kinase assay. A. Recombinant GST-ΔN LRRK2 phosphorylates recombinant human p53 WT (**A**) or mutant proteins **B**. The recombinant p53 protein was subjected to the *in vitro* kinase assay with cold ATP (b − d) or γ^32^P-ATP (a) and analyzed by Western blot with the indicated antibodies (b, p-TXR: phospho-TXR; c, LRRK2: MJFF2; d, p53:DO1) or autoradiography (a). WT: wild type, GS: G2019S, DA: D1994A. **C**. A schematic diagram of the p53 functional domains and location of the human p53 TXR motifs. Thr 387 which was used as a control was also shown. Numbers below the gel figures are the densitometric results for each band, [radiolabeled p53(a)]/[total p53(d)]

We were interested in identifying novel LRRK2 kinase substrates. Because of p53 activation in PD brains, we tested whether LRRK2 phosphorylates p53 using *E. coli*-expressed recombinant p53 protein and [γ-^32^P]ATP in an *in vitro* kinase assay. The GST-ΔN LRRK2 wild type (WT) phosphorylated p53 significantly and the corresponding G2019S (GS) and kinase-dead D1994A (DA) mutant proteins enhanced and nullified p53 phosphorylation, respectively, as expected (Fig. 1A-a). The GST-ΔN LRRK2 was used instead of the full length WT because the former exhibited stronger kinase activity than the latter (data not shown).

Because the TXR motif is suggested as a conserved LRRK2 phosphorylation site [20, 40–42], we checked whether such sites are present in p53. We found three sites: 211TFR, 304TKR, and 377TSR (numbers indicate position of threonine in human p53 protein, Fig. 1c). To investigate whether p53 is phosphorylated on these sites, we tested to see if phosphorylated p53 was detectable by Western blot with anti-phospho-TXR antibody (p-TXR). p-TXR is a commercial phospho-specific antibody that recognizes a phosphorylated threonine residue in the TXR motif. This antibody was shown to detect a specific phosphorylated threonine (T1410) in the LRRK2 TXR motif (TQR) after autophosphorylation of LRRK2 [41]. The Western blot result using the p-TXR antibody after the *in vitro* kinase assay detected phosphorylated p53 proteins after incubation with LRRK2 WT or GS, but not with DA. This is similar to the autoradiogram shown in Fig. 1A-a, suggesting that p53 was phosphorylated at threonines in the TXR sites (Fig. 1A-b). The *in vitro* kinase assay was carried out with p53 WT and the mutant proteins to detect the p53 phosphorylation sites. We first used the 1–293 deletion mutant of p53, in which the C-terminus of 100 amino acids has been deleted. As shown in Fig. 1B-b, the p-TXR antibody detected no signal in the 1–293 proteins after incubation with LRRK2, whereas it detected strong signal in the T387A, a positive control, suggesting that TXR phosphorylation sites are present on the C-terminal 100 amino acids. When we used the T304A, T377A, and T304/377A point mutant proteins in which the indicated threonine residues were mutated to alanine, the p-TXR antibody signal decreased in both T304A and T377A mutants and disappeared in the double-mutant T304/377A protein, suggesting that these sites are phosphorylated by LRRK2 (Fig. 1B-b). In contrast to the Western blot data, autoradiography revealed residual phosphorylation of the T304/377A protein, although radioactivity decreased by up to 50 % when compared to that of the WT (Fig. 1B-a). This residual activity was similar to the level observed in the 1–293 deletion protein, suggesting that although LRRK2 mainly phosphorylates p53 via the T304 and T377 sites, there may be additional LRRK2-mediated phosphorylation sites in the N-terminal 293 amino acid region (Fig. 1B-a). The kinase assay with the recombinant LRRK2 WT full length yielded a similar result (Additional file 1: Figure S1A). The p-TXR antibody specificity was confirmed by two separate results. First, p53 WT or the T304/377A double-mutant was not recognized by the p-TXR antibody (Fig. 1A-b & B-b). Second, moesin, an LRRK2 kinase substrate, was detected by the p-TXR antibody after the moesin was used as substrate in an *in vitro* kinase assay (Additional file 1: Figure S1B). Moesin has been reported to be phosphorylated by LRRK2 at T558 in TLR site by LRRK2 [40].

We applied MS to clearly determine the p53 phosphorylated residues. The p53 protein was used for the MS analysis with or without co-incubation of the LRRK2 G2019S recombinant proteins. However, two such trials failed to detect a positive signal.

### LRRK2 interacts with and phosphorylates p53 in physiological condition

**Fig. 2:**
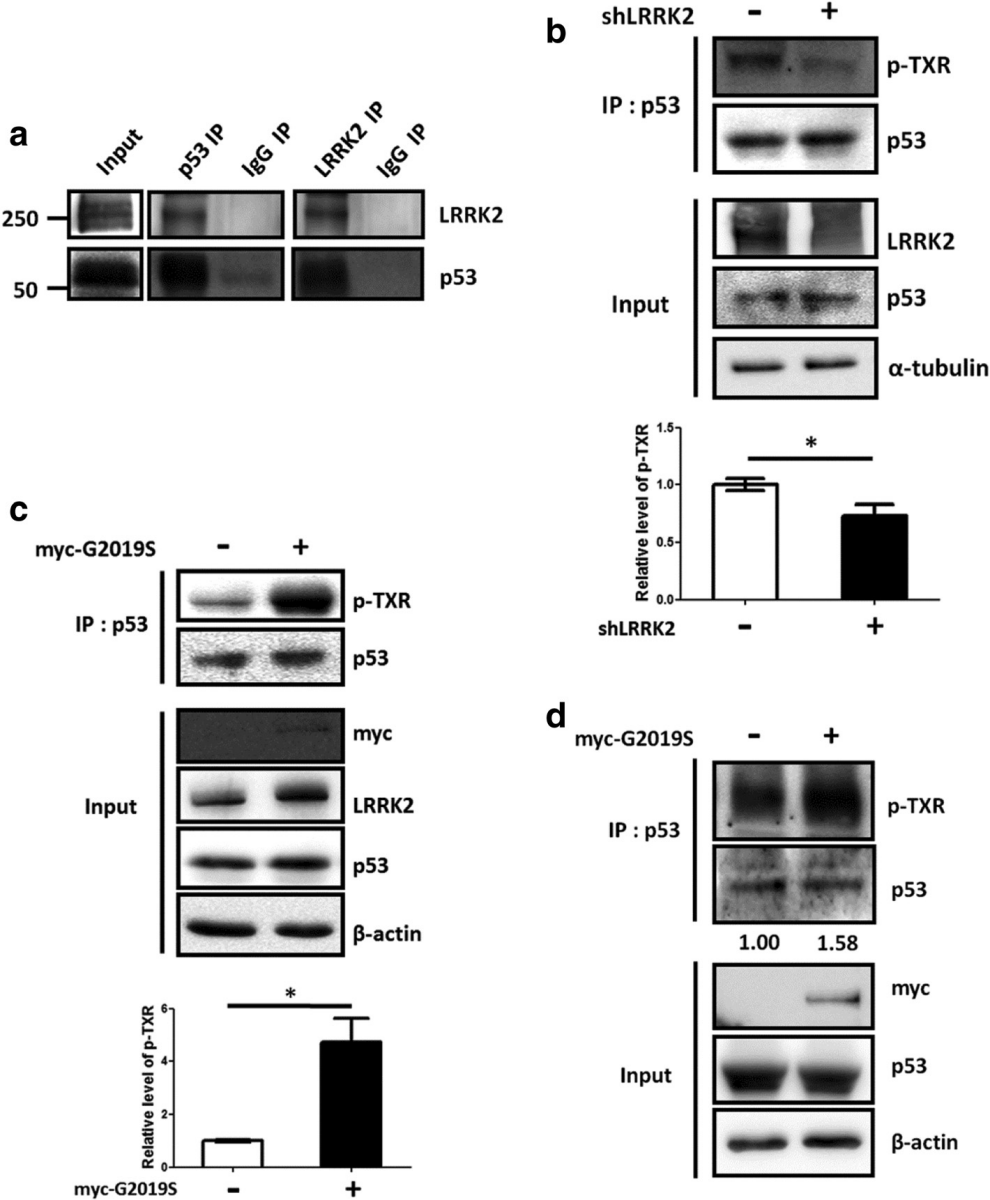
Phosphorylation of Thr in the p53 TXR sites is dependent on LRRK2 kinase activity. **a**. Interaction between LRRK2 and p53. Lysates from differentiated SH-SY5Y cell were immunoprecipitated with the indicated antibodies or control antibody, and each immunoprecipitate (p53IP, LRRK2IP or IgG IP) was subjected to the Western blot with the indicated antibodies. The experiments were carried out three times, and a representative image is shown. 20 % of input lysates are shown (Input). B & C. Phosphorylation level of Thr in the p53 TXR sites are proportional to LRRK2 expression level. Differentiated SH-SY5Y cells were transfected with the shLRRK2 (**b**) or myc-G2019S (**c**) plasmids for 18 or 24 h, respectively, and the cell lysates were immunoprecipitated with the p53 antibody. Both the immunoprecipitates (IP: p53) and 20 % or 10 % cell lysates (Input) of the shLRRK2 or G2019S transfection, respectively, were subjected to Western blot analysis with the indicated antibodies. – indicates transfection with the empty vector. D. Rat primary neurons over-expressing LRRK2 G2019S increased phosphorylation of Thr in the p53 TXR sites. The indicated cell lysates were immunoprecipitated with the p53 antibody and the immunoprecipitates (IP: p53) were subjected to Western blot. Input indicates 20 % of the cell lysates. The experiments were repeated three times, and a representative result is shown with a statistical analysis of the three results from each experiment, except D which was carried out once. Numbers below the gel figure (**d**) are relative protein level of each TXR band ([p-TXR]/[total p53]) based on the densitometric analysis. The antibodies used for Western blot analysis were indicated in the right side of each blot. *: *p* <0.05

Next, we tested whether LRRK2 and p53 interact with each other in physiological condition. The human neuroblastoma SH-SY5Y cell line is one of the most widely used dopaminergic cellular models with differentiation by retinoic acid. Moreover, treatment of cells with retinoic acid significantly increases LRRK2 expression [43] with little difference of p53 expression, increasing the possibility for LRRK2-mediated p53 phosphorylation (Additional file 2: Figure S2). The differentiated cell lysates were co-immunoprecipitated with the p53 or LRRK2 antibody. The results showed that both LRRK2 and p53 were specifically co-immunoprecipitated with the corresponding partner protein (Fig. 2a). To measure p53 cellular phosphorylation level by LRRK2, the cell lysates were immunoprecipitated with the p53 antibody and detected with the p-TXR antibody. Transfection of the SH-SY5Y cells with shLRRK2 or myc-G2019S plasmid significantly decreased or increased p-TXR levels in the p53 immunoprecipitates, respectively (Fig. 2b & c). The increase of p53 phosphorylation was also observed in the rat primary neuron cells overexpressing G2019S and dopaminergic neurons differentiated from human induced pluripotent stem (iPS) cells derived from fibroblasts of a G2019S carrier (Fig. 2d & Additional file 3: Figure S3). We conducted a similar experiment after treating the cells with LRRK2-IN-1, the LRRK2 kinase specific inhibitor [44]. Inhibitor treatment decreased p53 phosphorylation (Additional file 4: Figure S4A).

Altogether, these results suggest that LRRK2 phosphorylates the threonine in the p53 TXR motifs. However, the high residual activity (>50 %) observed in samples treated with LRRK2-IN-1 suggested that LRRK2 is not the sole kinase phosphorylating such p53 sites although we cannot exclude the possibility that the residual activity is due to incomplete inhibition of LRRK2 kinase activity in the LRRK2-IN-1 treatment (Additional file 4: Figure S4A, pS935 in the input panel). In fact, various kinases such as TFIIH CDK7-cycH-p36 [45], protein kinase C [46, 47], CHK1, and CHK2 [48] reportedly phosphorylate at least p53 Thr377.

### LRRK2-induced p53 phosphorylation accumulates p53 in the nucleus and activates p21^WAF1/CIP1^ expression

**Fig. 3:**
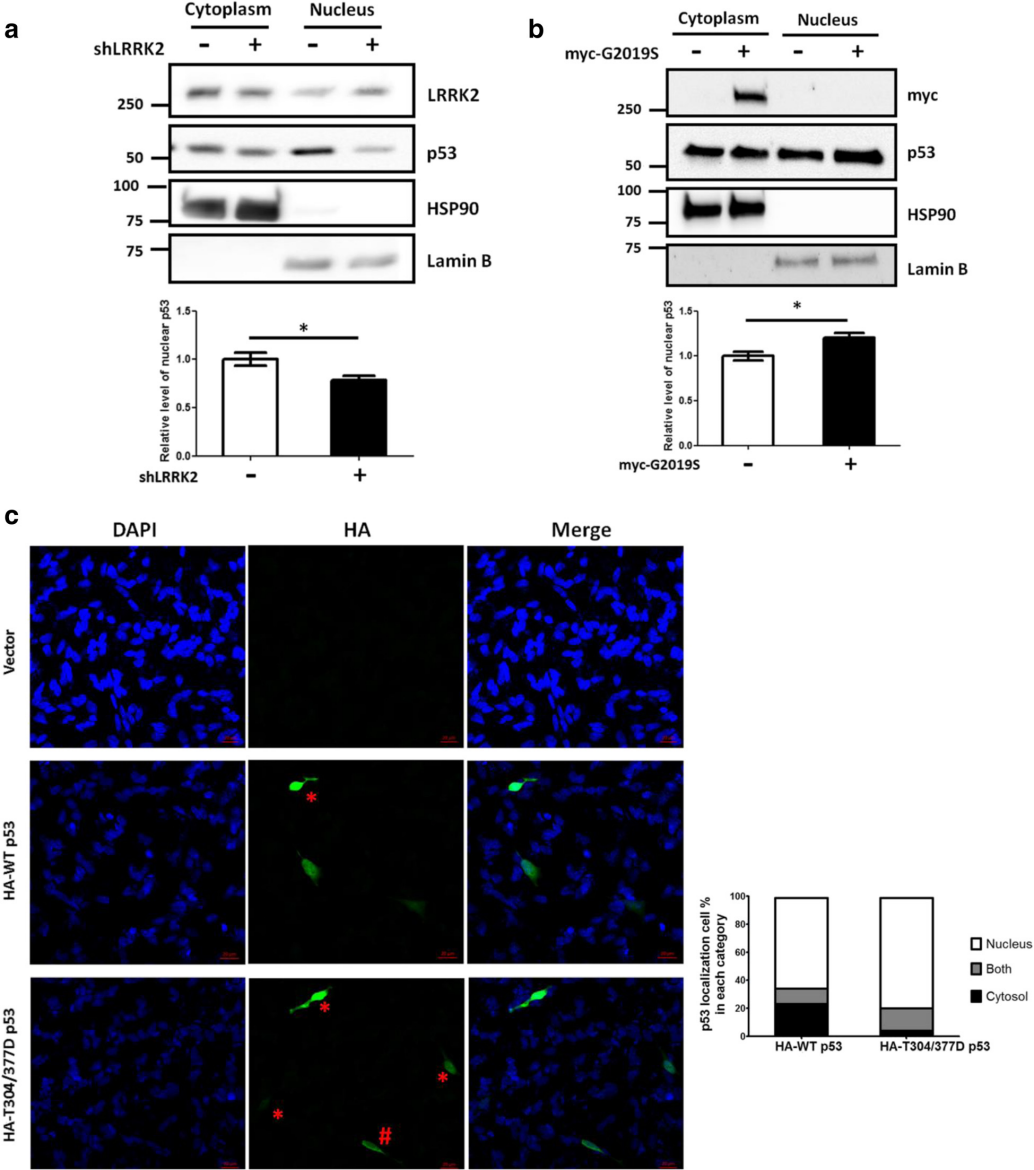
LRRK2 kinase activity regulates nuclear localization of p53. Down-regulation of LRRK2 expression by shLRRK2 transfection (**a**) or over-expressing G2019S (**b**) regulates the amount of nuclear p53. Differentiated SH-SY5Y cells were transfected with the indicated plasmids, the cell lysates were fractionated, and the cytoplasmic and nuclear fractions were subjected to the Western blot with the indicated antibodies. Lamin B and HSP90 were used for nuclear and cytoplasmic markers, respectively. (**c**) Nuclear localization of p53 protein was increased in the phosphomimetic T304/377D mutant compared to p53 WT. HA-tagged p53 WT or T304/377D plasmids were transfected to the SH-SY5Y cells, and the cells were immunostained with DAPI and Alexa 488 (HA). Ten images were taken and the transfected cells were chosen for the analysis (*n* = 3, Total cell numbers are 143 for the WT and 118 for the mutant). The localization of p53 protein was blindly determined as nuclear, cytoplasmic, or both, and the number of cells of each case was counted. Each representative image and the bar graph are shown. # and * indicate cells predominantly expressing cytoplasmic and nuclear p53, respectively. *: *p* <0.05

p53 is an important transcription factor. Human p53 contains three functional nuclear localization signals (NLSs), such as 305 ~ 322, 369 ~ 375, and 379 ~ 384([49], Fig. 1C). We tested whether the structural change, which may be caused by phosphorylating T304 and T377 sites, could affect p53 nuclear localization because they are very close to these NLSs. The SH-SY5Y cell line contains WT p53, which is located in the cytoplasm and nucleus [50]. Knocking down of LRRK2 expression by shLRRK2 transfection or ectopic expression of G2019S significantly decreased or increased the amount of nuclear p53 compared to that in the control, respectively (Fig. 3a & b). The treatment of LRRK2 kinase inhibitor exhibited a similar result to that of shLRRK2 transfection (Additional file 4: Figure S4B). These results suggest that LRRK2 kinase mediated p53 phosphorylation causes accumulation of nuclear p53. In addition, immunofluorescence staining assay after ectopic expression of the p53 WT or phosphomimetic mutant T304/377D proteins in the differentiated SH-SY5Y cell line also showed that the number of cells predominantly expressing nuclear p53 increased in cells transfected with the mutant p53 compared to those with the p53 WT (Fig. 3c).

**Fig. 4:**
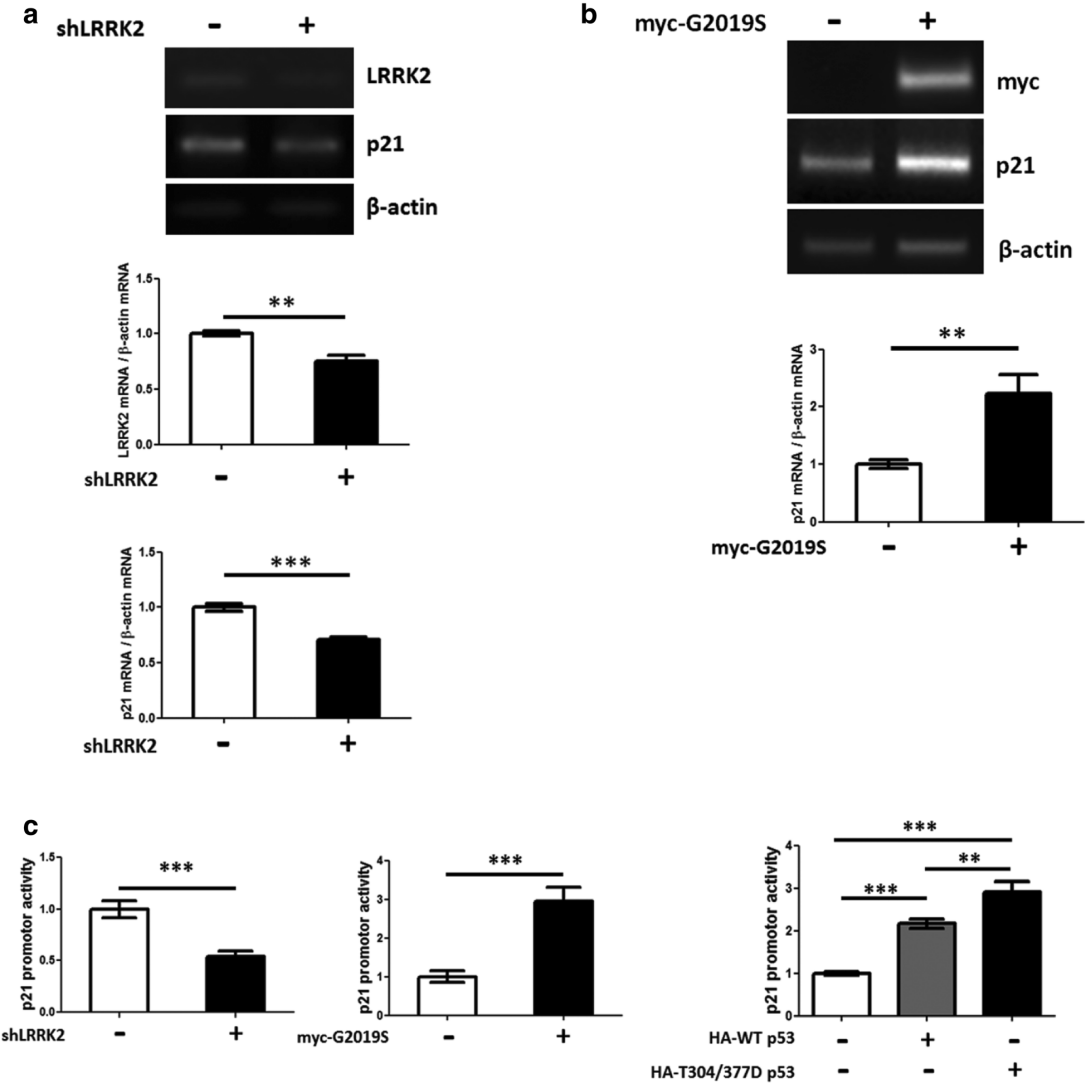
LRRK2 induces p21 transcription in differentiated SH-SY5Y cells. Differentiated SH-SY5Y cells were transfected with the shLRRK2 plasmid for 18 h (**a**) or the myc-tagged G2019S for 24 h (**b**). cDNA was synthesized from mRNA isolated from the cell lysates in each case. Semi-quantitative PCR was carried out using the cDNA as template, with the specific primers (Table 1) for the indicated genes. All experiments were repeated at least three times, and a representative result is shown with the statistical analysis. **c**. Activity of the p21 promoter-luciferase reporter from cells transfected with shLRRK2, G2019S mutant, or p53 WT and T304/377D. The experiment was done in nine separate wells in three sets. **: *p* <0.01; ***: *p* <0.001

**Fig. 5:**
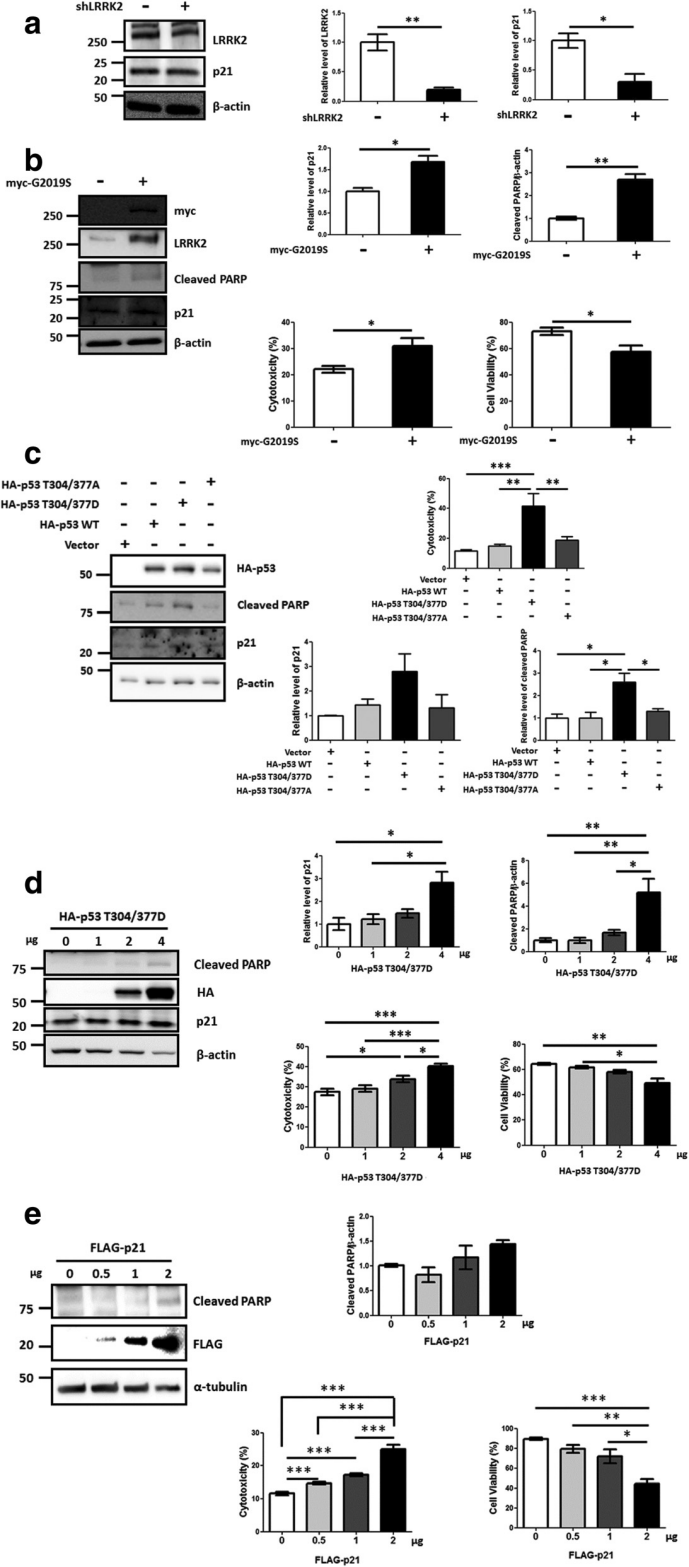
LRRK2 mediated p53 phosphorylation induces p21 expression and cytotoxicity in differentiated SH-SY5Y cells. The SH-SY5Y cells were transfected with the plasmid expressing shLRRK2 (**a**), G2019S (**b**), HA-p53, T304/377D and T304/377A (**c**), HA- T304/377D (D) or FLAG-p21(E). The p53 T304/377D mutant (**d**) or p21 (**e**) were transfected in a dose-dependent manner. The cell lysates were subjected to Western blot with the indicated antibodies with β-actin or α-tubulin as the loading controls. The transfected cells were also subjected to LDH (cytotoxicity) and/or CCK8 (Cell viability) assay (B ~ E). Actual transfection of each plasmid was confirmed by expression of the corresponding proteins. All experiments were repeated three times, and a representative Western image and a graphic representation with the statistical analysis are shown. *: *p* <0.05; **: *p* <0.01; ***: *p* <0.001

Next, we investigated the physiological consequences of LRRK2-induced p53 phosphorylation. We again utilized LRRK2-IN-1 and plasmids expressing shLRRK2 or G2019S. After each treatment or transfection, the cells were analyzed for p21^WAF1/CIP1^ (p21) expression by RT-PCR and Western blot. Transfection of the cells with shLRRK2 or G2019S resulted in decreased or increased p21 mRNA and protein levels, respectively (Figs. 4a & b and 5a & b). We also used reporter plasmids expressing a luciferase under control of the p21(waf) promoter to further confirm the regulation of p21 by LRRK2-mediated p53 phosphorylation at the transcriptional level [51]. The shLRRK2 transfection decreased luciferase activity, whereas overexpressing LRRK2 G2019S or the phosphomimetic p53 T304/377D increased it (Fig. 4c). In addition, we observed that G2019S-expressing cells significantly increased cleaved PARP, an apoptotic marker, and released lactate dehydrogenase (LDH) activity, and reduced cell viability in the CCK 8 assay, suggesting apoptosis-mediated cytotoxicity (Fig. 5b). Treating SH-SY5Y cells with a LRRK2 kinase inhibitor, LRRK2-IN-1, exhibited the similar results- decrease of p21 mRNA and protein levels based on the RT-PCR and Western blot assays (Additional file 4: Figure S4C-D). Treatment of the cells with GSK2578215A, another LRRK2 kinase specific inhibitor [52], also exhibited decrease of p21 expression (Additional file 4: Figure S4E). The effectiveness of LRRK2-IN-1 and shLRRK2 was confirmed by the reduction in phosphorylated S-935 LRRK2 [44] and LRRK2 mRNA and protein levels, respectively (Figs. 4a and 5a and Additional file 4: Figure S4A). Besides, exogenous expression of p53 WT, T304/377D and T304/377A exhibited increase of p21 expression, cleaved PARP and cytotoxicity in order of T304/377D > WT ≈ T304/377A > vector (Fig. 5c). Moreover, direct expression of the T304/377D or exogenous FLAG-p21 by transfection also exhibited similar results- increase of p21, cleaved PARP and cytotoxicity in a dose-dependent manner (Fig. 5d and e).

**Fig. 6:**
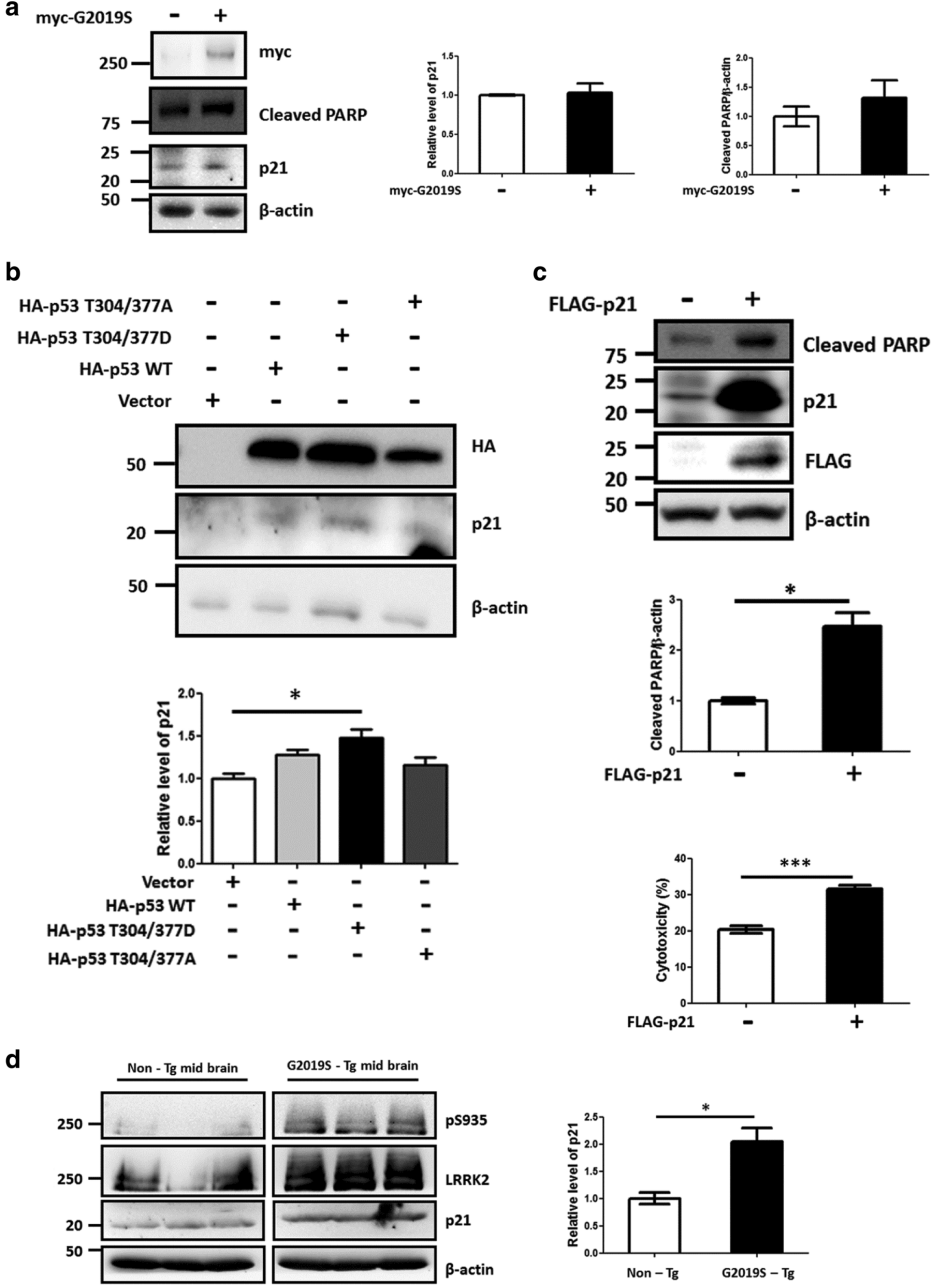
LRRK2 mediated p53 phosphorylation induces p21 expression in lysates of rat primary neurons and midbrain of G2019S TG mice. Transfection of rat primary neurons with myc-G2019S (**a**), HA-p53, T304/377D and T304/377A (**b**) or FLAG-p21(**c**) was carried out and expression levels of p21 were detected by Western blot analysis. The cells transfected with FLAG-p21 were also subjected to LDH assay to measure cytotoxicity (**c**). The experiments were repeated three (**a**, **c**) or four (**b**) times, and a representative result is shown with the statistical analysis. **d**. The midbrain lysates of G2019S TG or corresponding littermate mice were subjected to the Western blot with the indicated antibodies (*n* = 3). *: *p* <0.05; ***: *p* <0.001

In addition, the rat primary neurons transfected with plasmids expressing myc-G2019S, p53 T304/377D or FLAG-p21 also exhibited increase of p21, cleaved PARP and cytotoxicity (Fig. 6 and Additional file 5: Figure S5). Similar to the SH-SY5Y cells, exogenous expression of p53 WT, T304/377D and T304/377A in the rat primary neurons exhibited increase of p21 expression in order of T304/377D > WT ≈ T304/377A > vector (Figs. 5c vs. 6b). The Western blot with the G2019S TG mice mid-brain lysates also revealed that p21 expression in the TG mice significantly increased compared to that in the non TG littermates (Fig. 6d).

Altogether, these data suggested the activation of p21 and apoptotic cell death via specific p53 phosphorylation by LRRK2 kinase activity.

## Discussion

### p53 as a LRRK2 kinase substrate

We found here that LRRK2 phosphorylated p53 on T304 and T377 using a specific p-TXR antibody (Fig. 1). We also observed cellular interaction of LRRK2 with p53 (Fig. 2a). Using the unbiased proteomics approach (Table S1 in [19]), p53 has been previously reported as one of LRRK2 interacting proteins, which supports our data.

In contrast to the Western blot data, the *in vitro* isotope kinase assay showed that the T304/377A mutant was still phosphorylated, although radioactivity decreased considerably compared to that of the WT (Fig. 1A & B). Combined with the Western blot result, our data suggest that LRRK2 phosphorylates p53 at other sites in addition to T304 and T377. These sites may occur in the 293 amino acids N-terminal. In fact, the p53 N-terminus is heavily phosphorylated, resulting in different effects on p53 function [35]. Repeated MS analysis of phosphorylated p53 after incubation with LRRK2 failed to reveal such sites, and the identity of the putative additional site(s) remains unknown. Therefore, the T304/377D mutant may have only exhibited a partial effect on LRRK2-mediated p53 phosphorylation, although we used the mutant as a phosphomimetic protein in this study. Therefore, we also employed the treatment of LRRK2 kinase inhibitors, and transfection of G2019S or shLRRK2 plasmid regulating the LRRK2 expression level. Regardless of differences in experimental approaches, corresponding differences in p21 expression and cleaved PARP level have been commonly observed in the differentiated SH-SY5Y cells, rat primary neurons, G2019S TG mice brain lysates and dopaminergic neurons differentiated from human iPS cells of a G2019S carrier (Figs. 5 & 6), suggesting that apoptotic cell death by LRRK2 [53] was partially mediated through p53 phosphorylation.

The p53 phosphorylation system is very complex. One specific kinase phosphorylates multiple p53 sites, and the same residue can be phosphorylated by multiple kinases in response to diverse stressors [54]. Phosphorylation of p53 results in various phenotypes, depending on the site phosphorylated, such as the changes in binding affinity for a specific partner protein, the stability or DNA binding affinity which usually results in a change in p53 transcription activity [35]. The p53 Thr 377 is one of the minor phosphorylation sites by TFIIH CDK7-cycH-p36 [45] or protein kinase C [46, 47]. In addition, both CHK1 and CHK2 phosphorylated Thr377 and Ser378, and 377/378D phosphomimetic mutation reduced p53 Lys373 acetylation [48], suggesting a repressed p53 transcriptional activity. Our results suggest that LRRK2-mediated p53 phosphorylation increases nuclear localization of p53 (Fig. 3) and activates p21 expression (Figs. 4, 5 and 6). The discrepancy in the results [48] may be because each kinase phosphorylates other specific sites in addition to T377.

In contrast to T377, phosphorylation on Thr304 has been reported in endogenous p53 in human foreskin fibroblasts treated with the p53 activating chemical, etoposide [55]. However, physiological function of this phosphorylation is unknown.

### Physiological consequences of p53 phosphorylation by LRRK2

p53 is a well-characterized transcription factor activating multiple genes that mediate apoptosis and cell growth arrest in response to various genotoxic and non-genotoxic stressors. We initially tested the effect of LRRK2-induced p53 phosphorylation on its target genes, p21 and MDM2, by RT-PCR and Western blot analyses. The results suggest that LRRK2-induced p53 phosphorylation activates expression of p21 (Figs. 4, 5 and 6) but not that of MDM2. MDM2 expression was changed to a pattern similar to that of p21, under some conditions, but the difference was not significant (data not shown).

P21^WAF1/CIP1^ (p21) is a cell growth inhibitor activated by p53 [56]. The functions of p21 in cell proliferation and neuronal cell toxicity are controversial. The induction of p21 is required for neuron survival [57]. However, increased p21 expression induced by p53 phosphorylation inhibits proliferation of embryonic neuronal cells [58]. Another study reported that p53 induces p21 expression and apoptosis in neuroblastoma cells [59]. In addition, DNA-damaging drug treatment induces p53 and p21 expression and results in apoptosis in SH-SY5Y cells [60]. In our study, LRRK2-mediated p53 phosphorylation in differentiated SH-SY5Y cells and primary neurons significantly increased p21 expression and cytotoxicity (Figs. 4, 5 and 6). This result was repeatedly confirmed under various conditions, such as ectopic expression of phosphomimetic T304/377D or G2019S mutants, treatment of two different LRRK2 kinase inhibitors, down-regulation of LRRK2 expression level by shLRRK2 transfection, and G2019S TG brain lysates (Figs. 4, 5 and 6 and Additional file 4: Figure S4). More importantly, ectopic expression of p21 itself in differentiated SH-SY5Y cells and rat primary neurons suggested that p21 activation in these cells results in apoptosis (Figs. 5e & 6c). We also tested the effect of p53 T304/377D expression in the human colon cancer HCT116 p53 null cell line. In contrast to differentiated SH-SY5Y cells, no p21 expression was observed in the promoter and Western blot assays of the HCT cell line, suggesting that the effect we observed may be a cell-type, perhaps a neuron-specific one (data not shown).

Transfection of the phosphomimetic p53 T304/377D mutant exhibited increase of p21 expression whereas transfection of the T304/377A mutant that was unable to be phosphorylated showed lower p21 expression, similar to that of the WT (Figs. 5c & 6b). The reason why the T304/377A mutant still contains the activity similar to the WT might be because the T304/377A mutant can be still phosphorylated by other kinases at additional sites, like the WT.

Involvement of p53 in PD pathogenesis has been suggested in several reports, showing that the expression of p53 is higher in PD brains than in non-PD brains and that several PD-causing chemicals, such as rotenone and 6-hydroxydopamine, activate p53 [61–67]. In addition, rotenone induces upregulation of p53, p38, phospho-p38, and Bax in cultured PC12 cells and rats [67]. Several studies investigating PD-causative genes revealed that p53 is an important regulator of PD. For example, parkin and p53 reciprocally regulate each other in a cell-specific manner. p53 is a transcriptional activator of *Parkin* in cancer cells [68], and Parkin is a p53 transcriptional repressor in neuronal cells [62]. In addition, cytosolic p53 inhibits mitophagy mediated by Parkin in mouse hearts [69]. However, these transcriptional regulations were observed in a cell-specific manner [69], as in our data showing differences in p21 activation between the SH-SY5Y and HCT116 p53 null cell lines. Two other PD-recessive genes, DJ-1 and PINK1, also regulate p53 functions [37, 70].

Because p53 is one of the most important tumor suppressors, the physiological consequence of p53 phosphorylation by LRRK2 related to cancer is a critical question. Our Western data show increase in p21 and cleaved PARP protein levels and cytotoxicity of G2019S overexpressing cells. The low incidence of most cancers, excluding melanoma, in patients with PD has been well-documented in several epidemiological studies [71–73]. Our data provide a possible mechanism for this observation- i.e., increase of LRRK2 kinase activity inducing p53 phosphorylation and increasing p21 expression and cytotoxicity in most tissues- which is probably making cells more susceptible to p53-activating stimuli and evading cancer. However, a contradictory study showed that LRRK2 gene amplification causes a certain types of cancers, such as papillary renal and thyroid cancers [74]. In addition, there are contradictory reports about the effect of the G2019S mutation on the cancer [75, 76]. More studies are required to explain this discrepancy. In addition, it remains to be determined whether our observation is directly related to a PD pathogenic mechanism by a LRRK2 G2019S mutation and which stimuli activate LRRK2 kinase activity related to p53.

## Conclusions

LRRK2, a PD causative gene, phosphorylates p53 at T304 and T377 residues. Phosphorylation of p53 by LRRK2 increases p53 nuclear localization, expression of p21^WAF1/CIP1^, cleaved PARP level, and cytotoxicity in differentiated SH-SY5Y cells and rat primary neurons.

## Methods

### Plasmids and recombinant proteins

Plasmids expressing the HA-tagged human p53 gene (16434) and FLAG-p21 (16240) were purchased from Addgene (Cambridge, MA, USA) and shLRRK2 plasmid specifically inhibiting expression of endogenous human LRRK2 (TI202451) from ORIGENE (Rockville, MD, USA). The pET28 bacterial vector (Novagen, Darmstadt, Germany) expressing histidine tagged full length p53 [His-p53(1–393 amino acids)], and deleted p53, [His-p53(1–293 amino acids)], were previously reported [58]. The indicated p53 mutations were introduced by *in vitro* site-directed mutagenesis with proper primer pairs, and their sequences were confirmed by sequencing the full length of the cloned open reading frame. LRRK2 and its mutant plasmids were constructed as described previously [31, 77]. The recombinant LRRK2 WT, G2019S, and D1994A proteins with or without the N-terminal 970 amino acids were purchased from Invitrogen (Carlsbad, CA, USA). The following antibodies were used: LRRK2 (ab133474 [MJFF2]; Abcam, Cambridge, MA, USA, 1:1000), p53 (for human p53: sc-126, Santa Cruz Biotechnology, Dallas, TX, USA, 1:1000 and #2527S, Cell Signaling Technology, Danvers, MA, USA, 1:500; for mouse p53: sc-1312, Santa Cruz Biotechnology, 1:100; for immunoprecipitation of mouse p53: ab26, Abcam,), p21 (sc-397, Santa Cruz Biotechnology, 1:100), pS935 (ab133450, Abcam, 1:500), Lamin B (sc-6217, Santa Cruz Biotechnology, 1:500), LDH (sc-33781, Santa Cruz Biotechnology, 1:500), β-actin (sc-47778, Santa Cruz Biotechnology, 1:1000), p-TXR (#2351S, Cell Signal Technology, 1:500), hsp90 (610418, BD transduction Laboratories, Bedford, MA, USA, 1:5000) α-tubulin (T5168, Sigma-Aldrich, St. Louis, MO, USA, 1:2500).

The p-TXR antibody, which recognizes PKC-mediated phosphorylation motif [78] was used to detect phospo-p53 phosphorylated on the p-TXR motif threonine residue.

### Cell culture and transfection

Human SH-SY5Y cells were cultured in DMEM containing 10 % fetal bovine serum at 37 °C with 5 % CO_2_ and differentiated in medium containing *all*-*trans* retinoic acid (10 μM) for 6–7 days to obtain dopaminergic neuron-like properties.

Rat primary cortical neuron cultures were carried out as described previously [31]. The LRRK2 specific kinase inhibitors LRRK2 IN-1 (438193, Merck Millipore, Darmstadt, Germany) and GSK2578215A (4629, Tocris Biosciences, Bristol, United Kingdom) was added as indicated. Transfection of plasmid was carried out with Lipofectamine LTX (Invitrogen) as recommended by the manufacturer.

### *In vitro* kinase assay

We used the N-terminal truncated protein fused to GST or full length recombinant LRRK2 protein (Invitrogen), for the LRRK2 *in vitro* kinase assay. Protein (50 ng) was used for each kinase reaction. The indicated proteins were incubated in 20 μl kinase buffer [2.5 μCi of γ^32^P-ATP (BLU502; Perkin Elmer, Waltham, MA, USA), 50 μM ATP, 25 mM Tris–HCl (pH 7.5), 5 mM β-glycerol phosphate, 2 mM DTT, 0.1 mM NA_3_VO_4_ and 10 mM MgCl_2_] containing approximately 100 ng of the indicated proteins at 30 °C for 20 min. p53 WT or mutant proteins were expressed and purified from the *E. coli* BL21 strain. The samples were subjected to sodium dodecyl sulfate-polyacrylamide gel electrophoresis and the dried gel was analyzed by autoradiography.

### Reprogramming of human fibroblasts to induced pluripotent stem cells and their differentiation to dopaminergic neurons

Normal human fibroblast (IMR-90, ATCC) and Parkinson’s disease patient fibroblasts with LRRK2-G2019S mutation (#ND29492, Coriell Cell Repositories, Camden, NJ, USA) were reprogrammed to induced pluripotent stem cells (iPSCs) as described in previous report [79] with some modifications. In detail, the vectors were introduced using the Neon (Invitrogen) and the reprogramming medium was supplemented with 0.5 μM A83-01 (Tocris, Bristol, UK), 3 μM CHIR99021 (Tocris), and 0.2 mM sodium butyrate (Sigma).

The use of human cells and human pluripotent stem cells (hPSCs) were reviewed by institutional review board of KRIBB and we followed the guidelines in all experiments. The hPSCs were differentiated to neuroectodermal spheres (NES) as described previously [80]. For the dopaminergic neuronal differentiation, NESs that were cultured for four days after passaging were placed in dopaminergic neural patterning medium (DPM; DMEM/F12, N2 supplement (1:100), B27 supplement (1:50), 100 U/ml penicillin-streptomycin (all from Invitrogen), 100 ng/ml SHH, 100 ng/ml FGF8, 1 μM purmorphamine (Tocris), 3 μM CHIR99021 (Tocris) and 0.2 mM 2-Phospho-L-ascorbic acid (Sigma)) for four days. Patterned NESs were dissected by McIlwain tissue chopper and attached on matrigel-coated culture dishes. After four days, DPM was replaced by terminal differentiation medium (TDM; DMEM/F12, N2 supplement (1:100), B27 supplement (1:50), 100 U/ml penicillin-streptomycin, 20 ng/ml BDNF, 20 ng/ml GDNF, 0.5 mM dibutyryl-cAMP (Enzo, Farmingdale, NY, USA), and 0.2 mM 2-Phospho-L-ascorbic acid (Sigma)). All cytokines were obtained from Peprotech (Rocky Hill, NJ, USA). To get differentiated neurons, half of TDM was replaced every two days until 2 weeks.

The lysates of the differentiated neurons were immunoprecipitated with p53 antibody and were subjected to the western blot analysis with p-TXR antibody to investigate the level of phospho-Threonine at TXR of p53.

### Immunoprecipitation, nuclear fractionation and Western blot

The cell lysates were centrifuged at 16,000 × g for 10 min at 4 °C to remove insoluble material. The supernatants were precleared, then immunoprecipitated with anti-p53 or anti-LRRK2 (MJFF2, Abcam) at 4 °C for 8 h, and further incubated with protein-A agarose (Pierce, Rockford, IL,USA) for 24 h. The antibody-protein complexes were subjected to Western blot analysis using the indicated antibodies.

Cell nuclei were fractionated using a Nuclear/Cytosol Fractionation kit (K266-100, Bioscience, Milpitas, CA, USA) as suggested by the manufacturer, after the indicated treatment.

*G2019S* TG mice (#009609, Jackson Laboratory, Bar Harbor, ME, USA) and normal control littermates were sacrificed by cervical dislocation. Brain tissues were disrupted using a Dounce Homogenizer (10 strokes) and lysed by passing the extracts through a 22-gauge needle five times. The brain lysates were centrifuged at 16,000 × g for 30 min at 4 °C to remove insoluble material, and supernatants were used for further Western blot analysis.

The immunoprecipitated antibody-protein complexes, cell or brain lysates, or fractionated samples were subjected to Western blot analysis using the indicated antibodies.

### Luciferase assay and reverse transcription –polymerase chain reaction (RT-PCR)

Differentiated SH-SY5Y cells were transfected with the indicated reporter plasmids. The shLRRK2 plasmid was co-transfected with the reporter plasmid to knock down LRRK2 expression. The luciferase assay was conducted using the Dual Luciferase Assay kit (Promega, Madison, WI, USA). pRL-TK was co-transfected to normalize transfection efficiency.

Total RNA was isolated from the indicated cells, used for cDNA synthesis, and the target mRNAs were amplified by PCR using the cDNAs as templates and the indicated primer sets under the indicated conditions (Table 1). The PCR products were analyzed by agarose gel analysis.

**Table 1:** The primers used in the RT-PCR assays

Primer name	Gene	Direction	Sequence	TM (°C)	Product size(bp)
hP21-F	P21	Forward	GTTCCTTGGGAGCCGGAGC	60	417
hP21-R		Reverse	GGTACAAGACAGTGACAGGTC		
LRRK2-7232	LRRK2	Forward	GTCATGATGACAGCACAGC	65	201
LRRK2-7440		Reverse	CTCTTACTCAACAGATGTTCGTCTC		
pMycXhoI-R	Myc tag	Reverse	CTTCTGAGATGAGTTTTTGTTCCT CGAC	57.3	254^a^
b-actin-F	β-actin	Forward	CGCCGCCAGCTCACCATG	62.5	120
b-actin-R		Reverse	CACGATGGAGGGGAAGACGG		

The polymerase chain reaction was carried out at 95 °C for 1 min and 35 cycles of 95 °C for 30 s, Tm for 30 s, 72 °C for 30 s, and 72 °C for 5 min

^a^The pMycXhoI-R primer was used with LRRK2-7232 to detect expression of Myc-LRRK2 G2019S

*Bp* base pairs

### Confocal microscopy

Differentiated SH-SY5Y cells were transfected with HA-tagged p53 WT, T304/377D, or empty vector. After incubation, cell were sequentially immunostained with HA antibody and Alexa Fluoro 488 secondary antibody (Invitrogen) and the nuclei were counterstained with 4',6-diamidino-2-phenylindole (DAPI). The experiments were repeated to prepare three sets. Each set was observed under a Zeiss LSM700 confocal microscope and ten images were randomly taken. Predominant localization of p53 protein was scored blindly as nuclear, cytosol or both and the number of cells in each case was counted.

### Statistical analysis

All statistical analysis was carried out with the Prism5 program (GraphPad Software, La Jolla, CA, USA).

## Additional files

Additional file 1: Figure S1. LRRK2 phosphorylates p53 and moesin at TXR sites in the *in vitro* kinase assay. A. Phosphorylation of various p53 proteins by the Flag-tagged full length LRRK2 protein (Invitrogen) after the *in vitro* kinase assay. Phosphorylated p53 was detected by either autoradiography or Western blot with the p-TXR antibody. B. Western blot analysis after the *in vitro* kinase assay using moesin, various GST-ΔN-LRRK2 WT and mutant proteins, and cold ATP. (DOC 88 kb) Additional file 2: Figure S2. Differentiating SH-SY5Y cells with retinoic acid induces LRRK2 expression with little difference of p53. Indicated concentration of all- trans-retinoic acid was treated for two days. (DOC 65 kb) Additional file 3: Figure S3. Increased phosphorylation of Thr in the p53 TXR sites in dopaminergic neurons differentiated from fibroblast-derived iPS cells that were obtained from the human G2019S carrier (WT/GS) and non-carrier (WT/WT). The indicated cell lysates were immunoprecipitated with the p53 antibody and the immunoprecipitates (IP: p53) were subjected to Western blot. Input indicates 20% of the cell lysates. Numbers below the gel figures are relative protein level of each TXR band ([p-TXR]/[total p53]) based on the densitometric analysis. The antibodies used for Western blot analysis were indicated in the right side of each blot. (DOC 57 kb) Additional file 4: Figure S4. LRRK2 kinase inhibitor decreased p21 expression. Differentiated SH-SY5Y cells were treated with 1 μM LRRK2-IN-1 for 2 h (A-D) or 0.5 μM GSK2578215A (Tocris Biosciences, Bristol, United Kingdom) for 12 h (E). The p53 immunoprecipitates of the cell lysates were used to detect p-TXR level (A). The LRRK2-IN-1 treated cells were also used to determine relative amount of nuclear p53 (B) as in Fig. 3a. The cell lysates were used to detect p21 mRNA (C) and protein (D) levels as in Figs. 4 and 5. p21 expression level of GSK2578215A treated cells were also tested (E). Activities of LRRK2 kinase inhibitor treatments were confirmed by reduced level of LRRK2 p935 (pS935). – indicates vehicle treatment. Lamin B and LDH were used for nuclear and cytosolic markers, respectively. All experiments were repeated three times, and a representative result is shown with a bar graph. *: p <0.05; **: p <0.01. (DOC 188 kb) Additional file 5: Figure S5. Expression of T304/377D mutant increased cytotoxicity in rat primary neurons. The neuronal cells were transfected with vector, HA-p53, T304/377D and T304/377A. The cytotoxicity was measured by LDH assay. n = 6. **: p <0.01; ***: p <0.001. (DOC 49 kb)

## Abbreviations

LRRK2: Leucine-rich repeat kinase 2
PD: Parkinson’s disease
iPSCs: induced Pluripotent Stem Cells
p21: p21^WAF/CIP1^
RT-PCR: Reverse Transcription – Polymerase Chain Reaction
TXR: Threonine– X – Arginine
NLS: Nuclear Localization Signals
CHK1 and CHK2: Check Point Kinase1 and Check Point Kinase2
LDH: Lactate Dehydrogenase
PARP: Poly ADP-ribose Polymerase
TG: Transgenic
HSP90: Heat Shock Protein 90
hPSCs: human Pluripotent Stem Cells
MS: Mass Spectrometry
NES: Neuroectodermal Sphere
BDNF: Brain-Derived Neurotrophic Factor
GDNF: Glial cell-Derived Neurotrophic Factor
DAPI: 4', 6-diamidino-2-phenylindole

## References

[1] Martin I, Dawson VL, Dawson TM (2011). Recent advances in the genetics of Parkinson's disease. Annu Rev Genomics Hum Genet.

[2] Seol W (2010). Biochemical and molecular features of LRRK2 and its pathophysiological roles in Parkinson's disease. BMB Rep.

[3] Paisan-Ruiz C, Jain S, Evans EW, Gilks WP, Simon J, van der Brug M (2004). Cloning of the gene containing mutations that cause PARK8-linked Parkinson's disease. Neuron.

[4] Zimprich A, Biskup S, Leitner P, Lichtner P, Farrer M, Lincoln S (2004). Mutations in LRRK2 cause autosomal-dominant parkinsonism with pleomorphic pathology. Neuron.

[5] Gilsbach BK, Kortholt A (2014). Structural biology of the LRRK2 GTPase and kinase domains: implications for regulation. Front Mol Neurosci.

[6] Zabetian CP, Samii A, Mosley AD, Roberts JW, Leis BC, Yearout D (2005). A clinic-based study of the LRRK2 gene in Parkinson disease yields new mutations. Neurology.

[7] Smith WW, Pei Z, Jiang H, Dawson VL, Dawson TM, Ross CA (2006). Kinase activity of mutant LRRK2 mediates neuronal toxicity. Nat Neurosci.

[8] West AB, Moore DJ, Biskup S, Bugayenko A, Smith WW, Ross CA (2005). Parkinson's disease-associated mutations in leucine-rich repeat kinase 2 augment kinase activity. Proc Natl Acad Sci U S A.

[9] Greggio E, Jain S, Kingsbury A, Bandopadhyay R, Lewis P, Kaganovich A (2006). Kinase activity is required for the toxic effects of mutant LRRK2/dardarin. Neurobiol Dis.

[10] West AB, Moore DJ, Choi C, Andrabi SA, Li X, Dikeman D (2007). Parkinson's disease-associated mutations in LRRK2 link enhanced GTP-binding and kinase activities to neuronal toxicity. Hum Mol Genet.

[11] MacLeod D, Dowman J, Hammond R, Leete T, Inoue K, Abeliovich A (2006). The familial Parkinsonism gene LRRK2 regulates neurite process morphology. Neuron.

[12] Plowey ED, Cherra SJ, Liu YJ, Chu CT (2008). Role of autophagy in G2019S-LRRK2-associated neurite shortening in differentiated SH-SY5Y cells. J Neurochem.

[13] Orenstein SJ, Kuo SH, Tasset I, Arias E, Koga H, Fernandez-Carasa I (2013). Interplay of LRRK2 with chaperone-mediated autophagy. Nat Neurosci.

[14] Lee BD, Shin JH, Vankampen J, Petrucelli L, West AB, Ko HS (2010). Inhibitors of leucine-rich repeat kinase-2 protect against models of Parkinson's disease. Nat Med.

[15] Lobbestael E, Baekelandt V, Taymans JM (2012). Phosphorylation of LRRK2: from kinase to substrate. Biochem Soc Trans.

[16] Zhao J, Hermanson SB, Carlson CB, Riddle SM, Vogel KW, Bi K (2012). Pharmacological inhibition of LRRK2 cellular phosphorylation sites provides insight into LRRK2 biology. Biochem Soc Trans.

[17] Yun H, Heo HY, Kim HH, DooKim N, Seol W (2011). Identification of chemicals to inhibit the kinase activity of leucine-rich repeat kinase 2 (LRRK2), a Parkinson's disease-associated protein. Bioorg Med Chem Lett.

[18] Matta S, Van Kolen K, da Cunha R, van den Bogaart G, Mandemakers W, Miskiewicz K (2012). LRRK2 controls an EndoA phosphorylation cycle in synaptic endocytosis. Neuron.

[19] Martin I, Kim JW, Lee BD, Kang HC, Xu JC, Jia H (2014). Ribosomal protein s15 phosphorylation mediates LRRK2 neurodegeneration in Parkinson's disease. Cell.

[20] Yun HJ, Park J, Ho DH, Kim H, Kim CH, Oh H (2013). LRRK2 phosphorylates Snapin and inhibits interaction of Snapin with SNAP-25. Exp Mol Med.

[21] Yun HJ, Kim H, Ga I, Oh H, Ho DH, Kim J (2015). An early endosome regulator, Rab5b, is an LRRK2 kinase substrate. J Biochem.

[22] Gillardon F (2009). Leucine-rich repeat kinase 2 phosphorylates brain tubulin-beta isoforms and modulates microtubule stability - a point of convergence in Parkinsonian neurodegeneration?. J Neurochem.

[23] Xiong Y, Yuan C, Chen R, Dawson TM, Dawson VL (2012). ArfGAP1 is a GTPase activating protein for LRRK2: reciprocal regulation of ArfGAP1 by LRRK2. J Neurosci.

[24] Stafa K, Trancikova A, Webber PJ, Glauser L, West AB, Moore DJ (2012). GTPase activity and neuronal toxicity of Parkinson's disease-associated LRRK2 is regulated by ArfGAP1. PLoS Genet.

[25] Bailey RM, Covy JP, Melrose HL, Rousseau L, Watkinson R, Knight J (2013). LRRK2 phosphorylates novel tau epitopes and promotes tauopathy. Acta Neuropathol.

[26] Gloeckner CJ, Schumacher A, Boldt K, Ueffing M (2009). The Parkinson disease-associated protein kinase LRRK2 exhibits MAPKKK activity and phosphorylates MKK3/6 and MKK4/7, in vitro. J Neurochem.

[27] Imai Y, Gehrke S, Wang HQ, Takahashi R, Hasegawa K, Oota E (2008). Phosphorylation of 4E-BP by LRRK2 affects the maintenance of dopaminergic neurons in Drosophila. Embo J.

[28] Ohta E, Kawakami F, Kubo M, Obata F (2011). LRRK2 directly phosphorylates Akt1 as a possible physiological substrate: impairment of the kinase activity by Parkinson's disease-associated mutations. FEBS Lett.

[29] Su YC, Guo X, Qi X (1852). Threonine 56 phosphorylation of Bcl-2 is required for LRRK2 G2019S-induced mitochondrial depolarization and autophagy. Biochim Biophys Acta.

[30] Kim B, Yang MS, Choi D, Kim JH, Kim HS, Seol W (2012). Impaired inflammatory responses in murine Lrrk2-knockdown brain microglia. PLoS One.

[31] Shin N, Jeong H, Kwon J, Heo HY, Kwon JJ, Yun HJ (2008). LRRK2 regulates synaptic vesicle endocytosis. Exp Cell Res.

[32] Saha S, Guillily MD, Ferree A, Lanceta J, Chan D, Ghosh J (2009). LRRK2 modulates vulnerability to mitochondrial dysfunction in Caenorhabditis elegans. J Neurosci.

[33] May P, May E (1999). Twenty years of p53 research: structural and functional aspects of the p53 protein. Oncogene.

[34] Brooks CL, Gu W (2004). Dynamics in the p53-Mdm2 ubiquitination pathway. Cell Cycle.

[35] Dai C, Gu W (2010). p53 post-translational modification: deregulated in tumorigenesis. Trends Mol Med.

[36] Liang SH, Clarke MF (2001). Regulation of p53 localization. Eur J Biochem.

[37] Alves Da Costa C, Checler F (2011). Apoptosis in Parkinson's disease: is p53 the missing link between genetic and sporadic Parkinsonism?. Cell Signal.

[38] Checler F, Alves da Costa C (2013). p53 in neurodegenerative diseases and brain cancers. Pharmacol Ther.

[39] Kato I, Maita H, Takahashi-Niki K, Saito Y, Noguchi N, Iguchi-Ariga SM (2013). Oxidized DJ-1 inhibits p53 by sequestering p53 from promoters in a DNA-binding affinity-dependent manner. Mol Cell Biol.

[40] Jaleel M, Nichols RJ, Deak M, Campbell DG, Gillardon F, Knebel A (2007). LRRK2 phosphorylates moesin at threonine-558: characterization of how Parkinson's disease mutants affect kinase activity. Biochem J.

[41] Pungaliya PP, Bai Y, Lipinski K, Anand VS, Sen S, Brown EL (2010). Identification and characterization of a leucine-rich repeat kinase 2 (LRRK2) consensus phosphorylation motif. PLoS One.

[42] Angeles DC, Gan BH, Onstead L, Zhao Y, Lim KL, Dachsel J (2012). Mutations in LRRK2 increase phosphorylation of peroxiredoxin 3 exacerbating oxidative stress-induced neuronal death. Hum Mutat.

[43] Park JM, Ho DH, Yun HJ, Kim HJ, Lee CH, Park SW (2013). Dexamethasone induces the expression of LRRK2 and alpha-synuclein, two genes that when mutated cause Parkinson's disease in an autosomal dominant manner. BMB Rep.

[44] Deng X, Dzamko N, Prescott A, Davies P, Liu Q, Yang Q (2011). Characterization of a selective inhibitor of the Parkinson's disease kinase LRRK2. Nat Chem Biol.

[45] Lu H, Fisher RP, Bailey P, Levine AJ (1997). The CDK7-cycH-p36 complex of transcription factor IIH phosphorylates p53, enhancing its sequence-specific DNA binding activity in vitro. Mol Cell Biol.

[46] Youmell M, Park SJ, Basu S, Price BD (1998). Regulation of the p53 protein by protein kinase C alpha and protein kinase C zeta. Biochem Biophys Res Commun.

[47] Delphin C, Huang KP, Scotto C, Chapel A, Vincon M, Chambaz E (1997). The in vitro phosphorylation of p53 by calcium-dependent protein kinase C--characterization of a protein-kinase-C-binding site on p53. Eur J Biochem.

[48] Ou YH, Chung PH, Sun TP, Shieh SY (2005). p53 C-terminal phosphorylation by CHK1 and CHK2 participates in the regulation of DNA-damage-induced C-terminal acetylation. Mol Biol Cell.

[49] Liang SH, Clarke MF (1999). A bipartite nuclear localization signal is required for p53 nuclear import regulated by a carboxyl-terminal domain. J Biol Chem.

[50] Isaacs JS, Hardman R, Carman TA, Barrett JC, Weissman BE (1998). Differential subcellular p53 localization and function in N- and S-type neuroblastoma cell lines. Cell Growth Differ.

[51] Hur W, Rhim H, Jung CK, Kim JD, Bae SH, Jang JW (2010). SOX4 overexpression regulates the p53-mediated apoptosis in hepatocellular carcinoma: clinical implication and functional analysis in vitro. Carcinogenesis.

[52] Saez-Atienzar S, Bonet-Ponce L, Blesa JR, Romero FJ, Murphy MP, Jordan J (2014). The LRRK2 inhibitor GSK2578215A induces protective autophagy in SH-SY5Y cells: involvement of Drp-1-mediated mitochondrial fission and mitochondrial-derived ROS signaling. Cell Death Dis.

[53] Iaccarino C, Crosio C, Vitale C, Sanna G, Carri MT, Barone P (2007). Apoptotic mechanisms in mutant LRRK2-mediated cell death. Hum Mol Genet.

[54] Matsumoto M, Furihata M, Ohtsuki Y (2006). Posttranslational phosphorylation of mutant p53 protein in tumor development. Med Mol Morphol.

[55] DeHart CJ, Chahal JS, Flint SJ, Perlman DH (2014). Extensive post-translational modification of active and inactivated forms of endogenous p53. Mol Cell Proteomics.

[56] El-Deiry WS, Tokino T, Velculescu VE, Levy DB, Parsons R, Trent JM (1993). WAF1, a potential mediator of p53 tumor suppression. Cell.

[57] Poluha W, Poluha DK, Chang B, Crosbie NE, Schonhoff CM, Kilpatrick DL (1996). The cyclin-dependent kinase inhibitor p21 (WAF1) is required for survival of differentiating neuroblastoma cells. Mol Cell Biol.

[58] Park J, Oh Y, Yoo L, Jung MS, Song WJ, Lee SH (2010). Dyrk1A phosphorylates p53 and inhibits proliferation of embryonic neuronal cells. J Biol Chem.

[59] McKenzie PP, Guichard SM, Middlemas DS, Ashmun RA, Danks MK, Harris LC (1999). Wild-type p53 can induce p21 and apoptosis in neuroblastoma cells but the DNA damage-induced G1 checkpoint function is attenuated. Clin Cancer Res.

[60] Cui H, Schroering A, Ding HF (2002). p53 mediates DNA damaging drug-induced apoptosis through a caspase-9-dependent pathway in SH-SY5Y neuroblastoma cells. Mol Cancer Ther.

[61] Kang H, Shin JH (2015). Repression of rRNA transcription by PARIS contributes to Parkinson's disease. Neurobiol Dis.

[62] da Costa CA, Sunyach C, Giaime E, West A, Corti O, Brice A (2009). Transcriptional repression of p53 by parkin and impairment by mutations associated with autosomal recessive juvenile Parkinson's disease. Nat Cell Biol.

[63] Costa CA, Checler F (2011). Apoptosis in Parkinson's disease: Is p53 the missing link between genetic and sporadic Parkinsonism?. Cell Signal.

[64] Mogi M, Kondo T, Mizuno Y, Nagatsu T (2007). p53 protein, interferon-gamma, and NF-kappaB levels are elevated in the parkinsonian brain. Neurosci Lett.

[65] Nair VD (2006). Activation of p53 signaling initiates apoptotic death in a cellular model of Parkinson's disease. Apoptosis.

[66] Sunico CR, Nakamura T, Rockenstein E, Mante M, Adame A, Chan SF (2013). S-Nitrosylation of parkin as a novel regulator of p53-mediated neuronal cell death in sporadic Parkinson's disease. Mol Neurodegener.

[67] Wu F, Wang Z, Gu JH, Ge JB, Liang ZQ, Qin ZH (2013). p38(MAPK)/p53-Mediated Bax induction contributes to neurons degeneration in rotenone-induced cellular and rat models of Parkinson's disease. Neurochem Int.

[68] Zhang C, Lin M, Wu R, Wang X, Yang B, Levine AJ (2011). Parkin, a p53 target gene, mediates the role of p53 in glucose metabolism and the Warburg effect. Proc Natl Acad Sci U S A.

[69] Hoshino A, Mita Y, Okawa Y, Ariyoshi M, Iwai-Kanai E, Ueyama T (2013). Cytosolic p53 inhibits Parkin-mediated mitophagy and promotes mitochondrial dysfunction in the mouse heart. Nat Commun.

[70] Choi HK, Choi Y, Kang H, Lim EJ, Park SY, Lee HS (2015). PINK1 positively regulates HDAC3 to suppress dopaminergic neuronal cell death. Hum Mol Genet.

[71] Minami Y, Yamamoto R, Nishikouri M, Fukao A, Hisamichi S (2000). Mortality and cancer incidence in patients with Parkinson's disease. J Neurol.

[72] Bajaj A, Driver JA, Schernhammer ES (2010). Parkinson's disease and cancer risk: a systematic review and meta-analysis. Cancer Causes Control.

[73] Devine MJ, Plun-Favreau H, Wood NW (2011). Parkinson's disease and cancer: two wars, one front. Nat Rev Cancer.

[74] Looyenga BD, Furge KA, Dykema KJ, Koeman J, Swiatek PJ, Giordano TJ (2011). Chromosomal amplification of leucine-rich repeat kinase-2 (LRRK2) is required for oncogenic MET signaling in papillary renal and thyroid carcinomas. Proc Natl Acad Sci U S A.

[75] Agalliu I, San Luciano M, Mirelman A, Giladi N, Waro B, Aasly J (2015). Higher frequency of certain cancers in LRRK2 G2019S mutation carriers with Parkinson disease: a pooled analysis. JAMA Neurol.

[76] Allegra R, Tunesi S, Cilia R, Pezzoli G, Goldwurm S (2014). LRRK2-G2019S mutation is not associated with an increased cancer risk: a kin-cohort study. Mov Disord.

[77] Heo HY, Park JM, Kim CH, Han BS, Kim KS, Seol W (2010). LRRK2 enhances oxidative stress-induced neurotoxicity via its kinase activity. Exp Cell Res.

[78] Nishikawa K, Toker A, Johannes FJ, Songyang Z, Cantley LC (1997). Determination of the specific substrate sequence motifs of protein kinase C isozymes. J Biol Chem.

[79] Okita K, Matsumura Y, Sato Y, Okada A, Morizane A, Okamoto S (2011). A more efficient method to generate integration-free human iPS cells. Nat Methods.

[80] Chae JI, Kim J, Woo SM, Han HW, Cho YK, Oh KB (2009). Cytoskeleton-associated proteins are enriched in human embryonic-stem cell-derived neuroectodermal spheres. Proteomics.

