# Correction to: Leucine-Rich Repeat Kinase 2 (LRRK2) phosphorylates p53 and induces p21^WAF1/CIP1^ expression

**DOI:** 10.1186/s13041-017-0327-6

**Published:** 2017-10-06

**Authors:** Dong Hwan Ho, Hyejung Kim, Jisun Kim, Hyuna Sim, Hyunjun Ahn, Janghwan Kim, Hyemyung Seo, Kwang Chul Chung, Bum-Joon Park, Ilhong Son, Wongi Seol

**Affiliations:** 1 InAm Neuroscience Research Center, Sanbon Medical Center, College of Medicine, Wonkwang University, 321 Sanbon-ro, Gunposhi, Gyeonggido Republic of Korea; 2 Department of Molecular and Life Sciences, Hanyang University, Ansanshi, Gyeonggido Republic of Korea; 3 Stem Cell Research Center, Korea Research Institute of Bioscience and Biotechnology (KRIBB), 125 Gwahak-ro, Yuseong-gu, Daejeon, Republic of Korea; 4 Korea University of Science & Technology (UST), 113 Gwahak-ro, Yuseong-gu, Daejeon, Republic of Korea; 5 Department of Systems Biology, College of Life Science and Biotechnology, Yonsei University, Seodaemun-gu, Seoul, Republic of Korea; 6 Department of Molecular Biology, College of Natural Science, Pusan National University, Pusan, Republic of Korea; 7 Department of Neurology, Sanbon Medical Center, College of Medicine, Wonkwang University, 321 Sanbon-ro, Gunposhi, Gyeonggido Republic of Korea

## Correction to: Molecular brain (2015) 8:54 DOI 10.1186/s13041-015-0145-7

In the original publication of this article [1], published on 18 September 2015, it was noticed that the information on a plasmid expressing HA-tagged human p53 gene is wrong. In this Correction the incorrect and correct information on this gene are shown (and marked bold).

In the first paragraph of the Methods section, the plasmid expressing HA-tagged human p53 gene is described as followed:

**Plasmids expressing the HA-tagged human p53 gene (16434)** and FLAG-p21 (16240) were purchased from Addgene (Cambridge, MA, USA) and shLRRK2 plasmid specifically inhibiting expression of endogenous human LRRK2 (TI202451) from ORIGENE (Rockville, MD, USA).

The correct description of the plasmid expressing HA-tagged human p53 gene is:

**Ha-p53 WT was previously described** [2]. FLAG-p21 was purchased from Addgene (#16240, Cambridge, MA, USA) and shLRRK2 plasmid specifically inhibiting expression of endogenous human LRRK2 (TI202451) from ORIGENE (Rockville, MD, USA).
