# Supplementary figures and images for: Leucine-Rich Repeat Kinase 2 (LRRK2) phosphorylates p53 and induces p21WAF1/CIP1 expression
Source: Mol Brain. 2015 Sep 18;8:54. doi: 10.1186/s13041-015-0145-7 (PMC4575451; doi:10.1186/s13041-015-0145-7)

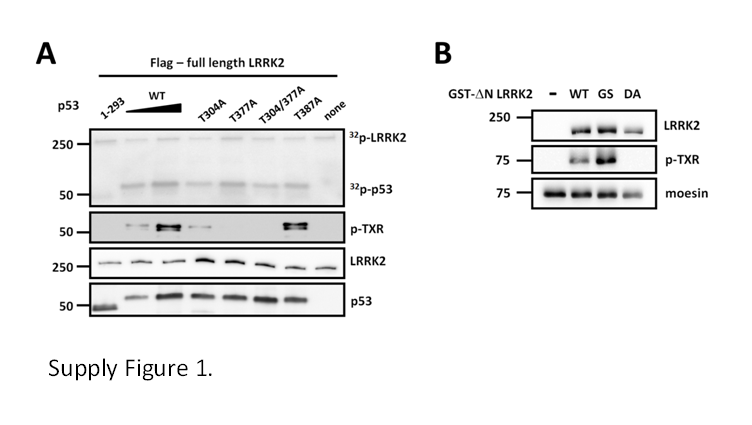

Supplement: Additional file 1: Figure S1. — LRRK2 phosphorylates p53 and moesin at TXR sites in the in vitro kinase assay. A. Phosphorylation of various p53 proteins by the Flag-tagged full length LRRK2 protein (Invitrogen) after the in vitro kinase assay. Phosphorylated p53 was detected by either autoradiography or Western blot with the p-TXR antibody. B. Western blot analysis after the in vitro kinase assay using moesin, various GST-ΔN-LRRK2 WT and mutant proteins, and cold ATP. (DOC 88 kb) [file 13041_2015_145_MOESM1_ESM.doc]

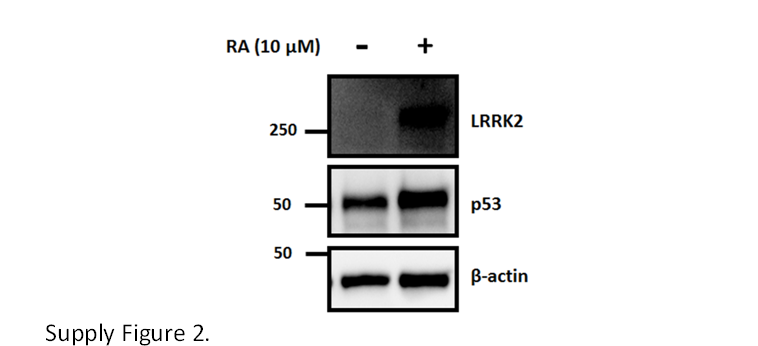

Supplement: Additional file 2: Figure S2. — Differentiating SH-SY5Y cells with retinoic acid induces LRRK2 expression with little difference of p53. Indicated concentration of all- trans-retinoic acid was treated for two days. (DOC 65 kb) [file 13041_2015_145_MOESM2_ESM.doc]

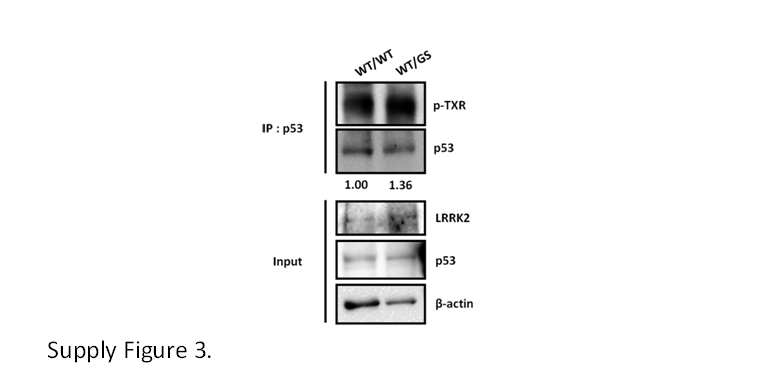

Supplement: Additional file 3: Figure S3. — Increased phosphorylation of Thr in the p53 TXR sites in dopaminergic neurons differentiated from fibroblast-derived iPS cells that were obtained from the human G2019S carrier (WT/GS) and non-carrier (WT/WT). The indicated cell lysates were immunoprecipitated with the p53 antibody and the immunoprecipitates (IP: p53) were subjected to Western blot. Input indicates 20% of the cell lysates. Numbers below the gel figures are relative protein level of each TXR band ([p-TXR]/[total p53]) based on the densitometric analysis. The antibodies used for Western blot analysis were indicated in the right side of each blot. (DOC 57 kb) [file 13041_2015_145_MOESM3_ESM.doc]

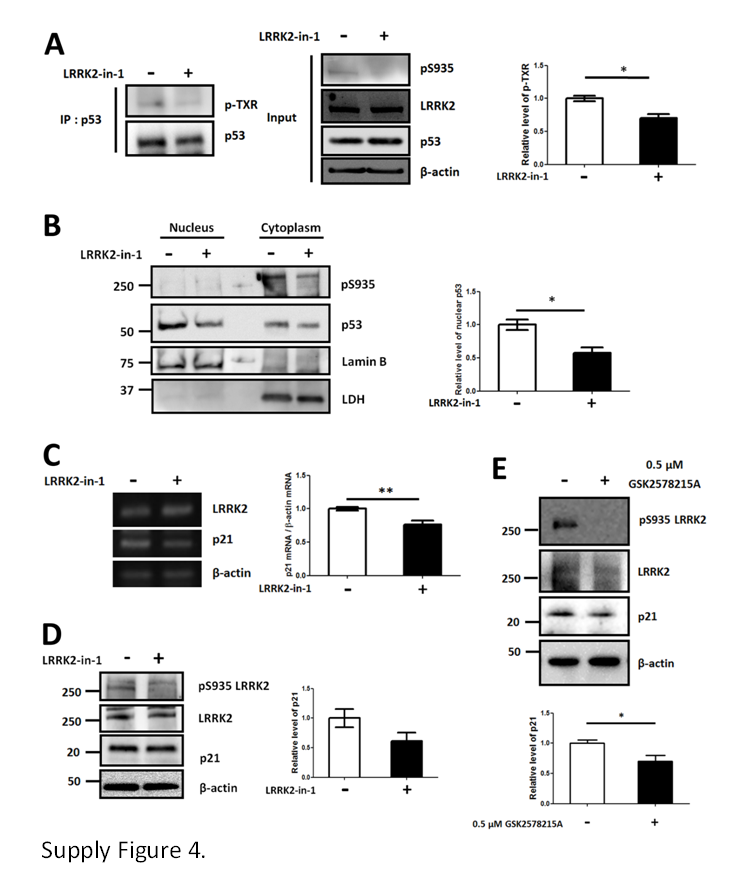

Supplement: Additional file 4: Figure S4. — LRRK2 kinase inhibitor decreased p21 expression. Differentiated SH-SY5Y cells were treated with 1 μM LRRK2-IN-1 for 2 h (A-D) or 0.5 μM GSK2578215A (Tocris Biosciences, Bristol, United Kingdom) for 12 h (E). The p53 immunoprecipitates of the cell lysates were used to detect p-TXR level (A). The LRRK2-IN-1 treated cells were also used to determine relative amount of nuclear p53 (B) as in Fig. 3a. The cell lysates were used to detect p21 mRNA (C) and protein (D) levels as in Figs. 4 and 5. p21 expression level of GSK2578215A treated cells were also tested (E). Activities of LRRK2 kinase inhibitor treatments were confirmed by reduced level of LRRK2 p935 (pS935). – indicates vehicle treatment. Lamin B and LDH were used for nuclear and cytosolic markers, respectively. All experiments were repeated three times, and a representative result is shown with a bar graph. *: p <0.05; **: p <0.01. (DOC 188 kb) [file 13041_2015_145_MOESM4_ESM.doc]

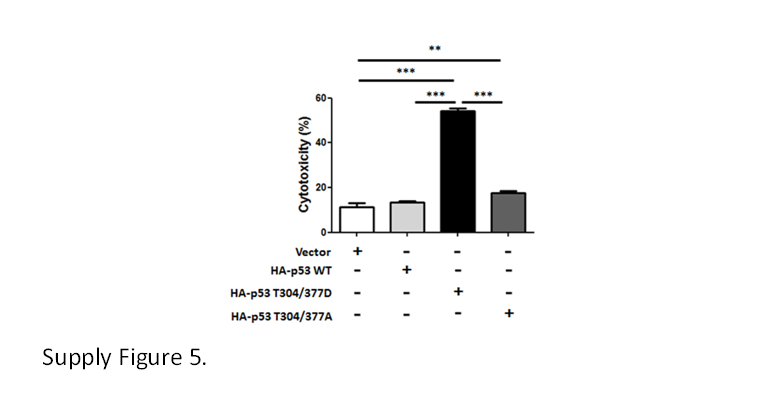

Supplement: Additional file 5: Figure S5. — Expression of T304/377D mutant increased cytotoxicity in rat primary neurons. The neuronal cells were transfected with vector, HA-p53, T304/377D and T304/377A. The cytotoxicity was measured by LDH assay. n = 6. **: p <0.01; ***: p <0.001. (DOC 49 kb) [file 13041_2015_145_MOESM5_ESM.doc]
